# Primary extranodal natural Killer/T-cell lymphoma in a child in the colon: A case report

**DOI:** 10.1097/MD.0000000000024232

**Published:** 2021-01-22

**Authors:** Yi Duan, Juan Huang, Johannes Haybaeck, ZhiHui Yang

**Affiliations:** a Department of Pathology, The Affiliated Hospital of Southwest Medical University, Luzhou, China; b Department of Pathology, Neuropathology, and Molecular Pathology, Medical University of Innsbruck, Innsbruck, Austria; c Diagnostic & Research Center for Molecular BioMedicine, Institute of Pathology, Medical University of Graz, Graz, Austria.

**Keywords:** childhood, extranodal natural Killer/T-cell lymphoma, GI tract

## Abstract

**Rationale:**

Primary extranodal natural killer (NK)/T-cell lymphoma (ENKTL) rarely occurs in childhood and adolescence. To the best of our knowledge, ENKTL of childhood in the gastrointestinal (GI) tract has not been reported yet.

**Patient concerns:**

A 12-year-old Chinese boy complained of abdominal pain and persistent fever for 1 month.

**Diagnosis:**

Grossly an ulcerated tumor with perforation was located at the proximal ascending colon, 5 cm × 4 cm × 1.5 cm in diameter. The tumor was poorly circumscribed, tan-white and solid. Histological evaluation revealed medium-sized atypical lymphoid cells with large areas of necrosis distributed throughout all layers of the colon. Small blood vessels with destroyed walls were surrounded by lymphoid cells. Immunohistochemistry (IHC) highlighted tumor cells as strongly positive for CD3, CD56, CD5, CD2, CD8, CD4, CD43, T-cell restricted intracellular antigen 1 (TIA-1) and granzyme B. The proliferation index, measured by Ki-67 expression was high with 60%. The In situ hybridization (ISH) for EBER was positive. TCR was negative. Therefore, the final diagnosis was ENKTL of childhood in the colon.

**Interventions:**

The patient underwent right hemicolectomy and ileocolostomy.

**Outcomes:**

We recommended further evaluation and treatment, but the patient and patient family rejected further treatment of his condition. The patient died within 1 month after being discharged from hospital as a result of his disease.

**Lessons:**

ENKTL of childhood in the GI tract is extremely rare. Due to the non-specific clinical symptoms, it is easy it is easy not to think of this differential diagnosis at early stage. If patients have GI symptoms, ENKTL cannot easily be ignored. It is necessary to diagnose ENKTL of childhood in the GI tract by morphology and immunohistochemistry, and to differentiate from the GI T-cell lymphomas. We hope this case may serve as a reference improving clinical diagnosis and treatment.

## 1 Introduction

Extranodal natural Killer/T-cell lymphoma (ENKTL) is a recognized distinct clinicopathologic entity that accounts for 3% to 8% of all lymphomas.^[1,2]^ According to the current World Health Organization (WHO) classification of lymphomas, ENKTL are divided into 2 subtypes, nasal and non-nasal type.^[3]^ The nasal type most commonly occurs in the nasopharyngeal area, the non-nasal type occurs in other areas including the gastrointestinal (GI) tract, skin, lung, testis and muscle. At present, ENKTL of the GI tract is relatively scarce, as it accounts for only 7% of all NK/T cell lymphomas.^[2]^ Their main symptoms present as abdominal pain, vomiting, and bowel perforation. Meanwhile, more than 60% of these patients present at advanced disease with B symptoms, including fever, night sweat, and weight loss.^[4,5]^ Importantly, ENKTL is known to occur commonly in adults, with a median age of 40 to 50 years.^[1]^ It is rarely observed in children and adolescents. Only a few of these cases were reported.^[6,7]^ The most commonly involved site was the upper aerodigestive tract. The clinicopathological features and outcomes in pediatric patients with ENKTL of the GI tract have never been reported before.

Here, we describe a unique case of primary ENKTL in a 12-year-old boy in the colon and explore its clinicopathological features, diagnostic criteria, differential diagnosis and prognosis.

## 2 Case report

A 12-year-old boy presented with a 1-month history of abdominal pain and persistent fever. He had no weight loss at presentation. The patient was treated with empirically selected antibiotics such as cefuroxime at the local hospital. However, his symptoms did not disappear. The patient had no family history of malignant tumors. Laboratory examinations revealed mild leukocytosis (16.39 × 10^9^/L), increased C-reactive protein (CRP) level (62.08 mg/L) and mild hypoalbuminemia (36.5 g/L). No bloody stool occurred. Physical examination revealed an abdominal mass, pain and tenderness in the right lower quadrant.

**Figure 1:**
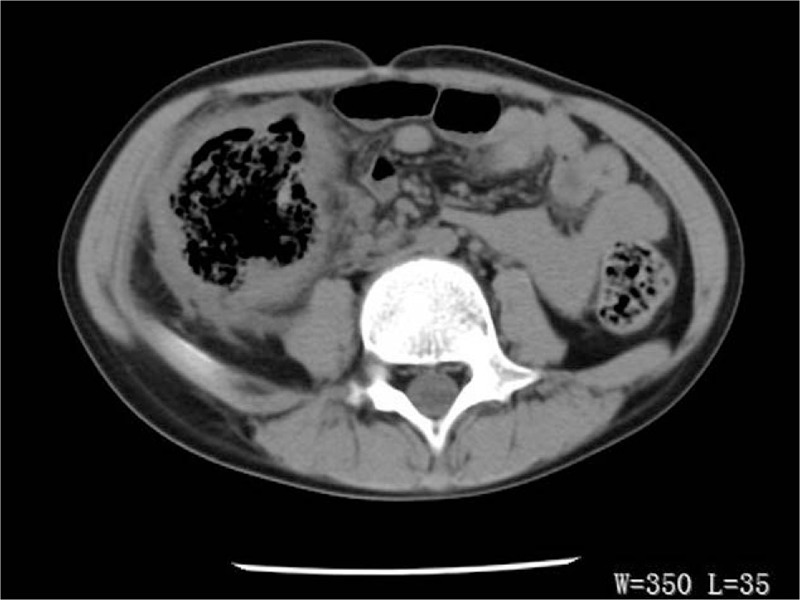
Computed tomography imaging demonstrated the bowel-wall thickening and luminal dilatation in the ascending colon.

Subsequently, abdominal computed tomography (CT) imaging demonstrated the bowel-wall thickening and luminal dilatation in the ascending colon, and localized peritonitis (Fig. 1). Accordingly, the patient was preliminarily considered as appendicitis with perforation. He underwent emergency surgery. During the surgery, a 5 cm in diameter large, ulcerated mass with perforation into the proximal ascending colon was found, the intestinal wall around the mass was edematous, and the intestinal cavity was dilated. A right hemicolectomy and ileocolostomy was performed. Lymphadenopathy and other organ mass were not identified.

Grossly, an ulcerated tumor with perforation was located at the proximal ascending colon, 5 cm × 4 cm × 1.5 cm in diameter. The tumor was poorly circumscribed, with a tan-white and solid cut surface. It invaded the entire intestinal wall. Enlarged lymph nodes were not detected.

**Figure 2:**
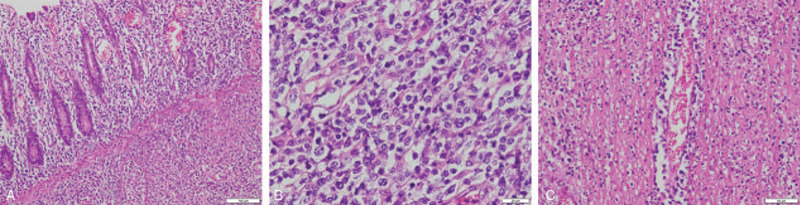
Microscope findings of the ascending colonic mucosa biopsy. (A) The medium-sized atypical lymphoid cells with large areas of necrosis were distributed over all layers of the colon (HE stain, 100 × magnification). (B) The medium-sized tumor cells were presented as irregular nuclear contours, condensed chromatin, inconspicuous nucleoli, and pale cytoplasm (HE stain, 400 × magnification). (C) Small blood vessels, which walls were destroyed, were found around by these lymphoid cells (HE stain, 200 × magnification).

**Figure 3:**
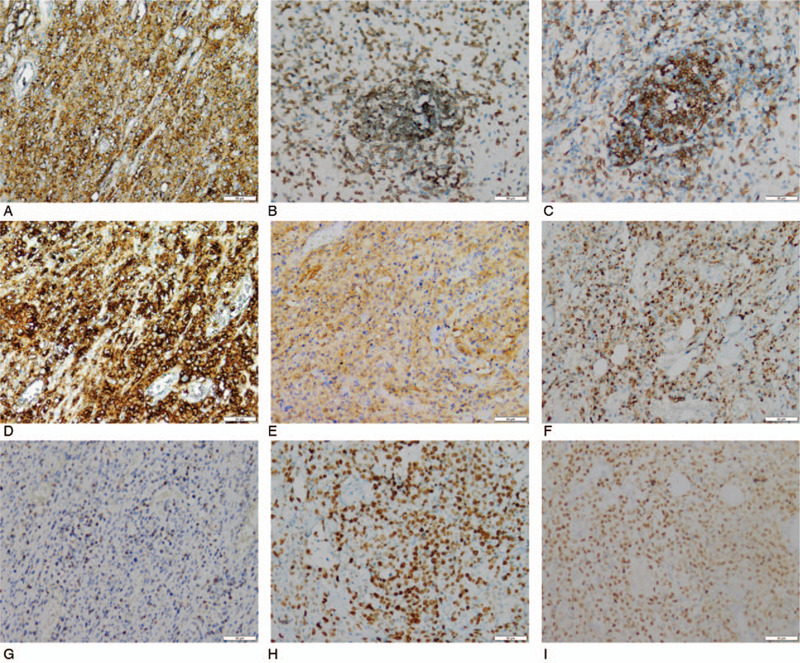
Immunohistochemical staining pattern of primary colonic NK/T-cell lymphoma. (A-G) The tumor cells were positive for CD2, CD3, CD4, CD7, CD56, granzyme B, and TIA-1 (IHC stain, 200 × magnification). (H) The tumor cells expressed Ki-67 with a high rate at 60% (IHC stain, 200 × magnification). (I) ISH for EBVR was positive (200 × magnification).

Histologically, the medium-sized atypical lymphoid cells with large areas of necrosis were distributed over all layers of the colon (Fig. 2A). These lymphoid cells were presented with irregular nuclear contours, condensed chromatin, inconspicuous nucleoli, and pale cytoplasm (Fig. 2B). Significantly, small blood vessels with destroyed walls and surrounded by lymphoid cells were observed (Fig. 2C). Immunohistochemistry (IHC) showed strongly positive CD3, CD56, CD5, CD2, CD8, CD4, CD43, TIA-1 and granzyme B tumor cells. A Ki-67 rate of 60% indicated a highly aggressive neoplasm (Fig. 3A-3H). Tumor cells were negative for CD20, CD30, and TCRβF1. In situ hybridization (ISH) showed EVBR was positive (Fig. 3I). TCR gene rearrangement was negative.

The final diagnosis was ENKTL, non-nasal type. According to the Ann Arbor system, the patient was staged as IIB. We recommended further evaluation and treatment, but the patient and his family rejected further treatment of his condition. The results of laboratory examinations returned to normal when the patient discharged. The patient died within 1 month after discharged from hospital.

## 3 Discussion

ENKTL is a distinct lymphoma that expresses cytotoxic phenotypes and has an association with Epstein-Barr virus (EBV) infection.^[8]^ It is more prevalent in Asian populations, especially in Southern China and Southeast Asia. It is a subtype of non-Hodgkin's lymphoma (NHL), occupies approximately 2% to 10%.^[9,10]^ The most commonly occurrence site of ENKTL is the nasal cavity and its adjacent parts (approximately 60%–90%), rarely in extra-nasal locations. Recent studies have shown the GI tract as an unusual site of occurrence. ENKTL of the GI tract accounts for only 3% of all primary GI non-Hodgkin lymphomas ^[11]^ and 7% of all NK/T cell lymphomas.^[2]^ Furthermore, ENKTL of the colon is very rare.

Primary ENKTL is known to occur commonly in middle age people. Only a few cases of ENKTL occurring in children and adolescents have been reported.^[6,7,12]^ It tends to have a male predominance and the clinical symptoms are not specific. Most patients present as stage I/II and frequently with B symptoms. Additionally, ENKTL commonly occurs in the nasal cavity and/or paranasal region,^[7]^ extremely rarely in the skin, orbit, liver, spleen and lung.^[6,12]^ Up to now, ENKTL of children and adolescents, occurring in the GI tract was never reported before. To the best of our knowledge, this is the first reported case of ENKTL of childhood in the GI tract.

The main symptom of GI NK/T-cell lymphoma is abdominal pain which can be associated with GI hemorrhage, vomiting, and bowel perforation.^[4,13]^ Some patients can accompany with weight loss and irregular fever. Radiological imaging examination demonstrated the diffuse thickening of the intestinal wall.^[13,14]^ The clinical symptom and radiological imaging of GI NK/T cell lymphoma is non-specific. It may mimic many other benign or malignant lesions. Because of these nonspecific clinical features, ENKTL can easily be ignored in the early stage. According to the clinical symptoms, one of the main differential diagnose for this patient is an inflammatory of abdomen. In our case, it has been misdiagnosed as appendicitis with perforation clinical. So, if patients have gastrointestinal hemorrhage, vomiting, bowel perforation, weight loss and irregular fever symptoms, ENKTL cannot be ruled out with certainty. Especially in case of GI tract biopsies, pathologists should pay more attention to observe the morphologic features to find the lymphoid cells, coagulative necrosis and an angiocentric and angiodestructive growth pattern, in order to avoid missed diagnosis of ENKTL.

The diagnosis of ENKTL in children and adolescents requires careful observation of the morphology and relevant use of ancillary studies. The morphologic features and immunophenotype are essential for the correct diagnosis. They are similar to that occurring in adults.^[6]^ Histologically, ENKTL is composed of small to medium-sized lymphoid cells with pale cytoplasm and basophilic particles. The presence of large coagulative necrosis, anginocentricity and anginodestruction are associated typical features of this tumor. Immunohistochemically, typical markers are CD2+, CD56+, and cytoplasmic CD3ε+. CD56 is a recognized NK-cell marker, presented in 60% to 100% of cases. CD2 and CD3 are T cell-associated antigens. Cytotoxic molecules, including granzyme B, TIA-1, and perforin are positive. CD20, a B-cell marker, is consistently negative. Virtually all cases of ENKTL are associated with EBV infection, proven either by elevated plasma EBV DNA levels or ISH for EBVR (Table 1).^[15]^ In addition, it is conducive to identify T cell lymphoma by TCR gene rearrangement. TCR gene rearrangement of ENKTL is not necessary.

**Table 1:** the clinical and pathological features about extranodal natural Killer/T-cell lymphoma and its differential diagnosis.

Disease	Clinical features	Morphologic features	Immunophenotype	ISH of EBVR
Extranodal natural Killer/T-cell lymphoma	ENKTL commonly occurs in the nasal cavity and/or paranasal region, extremely rarely in the skin, orbit, liver, spleen and lungMost patients present as stage I/II and frequently with B symptoms.	Small to medium-sized lymphoid cellslarge coagulative necrosisanginocentricity anginodestruction	CD56+, CD3ε+, CD2+, granzymeB+, TIA-1+, perforin+	positive
Enteropathy-associated T cell lymphoma	A lethal type of T cell lymphomaCeliac disease	uniformly large-sized lymphoid cells	CD3+, CD2+, CD5+, CD8+CD56-	negative
Systemic EBV^+^ T cell lymphoma of childhood	Acute onset with EBV infectionQuickly multi-organ failureHPS	small-sized lymphoid cells with mild atypiahistiocytes phagocytizing red blood cells	CD8+, TIA-1+CD56-	positive
Chronic active EBV disease	Infectious mononucleosis-like symptoms for more than 3 monthsElevation of plasma EBV DNA	nonspecific inflammationthe absence of the malignant lymphoproliferation	CD4+, CD8+CD56-	positive

When ENKTL in children and adolescents occurs in the GI tract, it needs to be differentiated from the following diseases: Enteropathy-associated T cell lymphoma (EATL), systemic EBV^+^ T cell lymphoma of childhood, and chronic active EBV disease (CAEBV). Enteropathy-associated T cell lymphoma (EATL), a lethal type of T cell lymphoma, commonly occurs in small intestine. Its clinical symptom is celiac disease similar to the ENKTL in the GI tract, but their morphologic features and immunophenotype are different.^[16]^ Histologically, EATL is composed of uniformly large-sized lymphoid cells. However, EATL is absent of perivascular growth of tumor cells and invades the vascular wall, which are typical morphologic features of ENKTL. Immunohistochemically, the tumor cells of EATL are positive with antibodies directed against CD3, CD2, CD5, and CD8, but negative with CD56. The disease is not related to an EBV infection. TCR indicates a clonal rearrangement of the sample (Table 1).^[17,18]^ Another disease that needs to be differentiated from ENKTL in children and adolescents is systemic EBV^+^ T cell lymphoma of childhood, which also occurs in adolescents. In the clinical manifestation, this disease has acute onset with EBV infection, and quickly causes multi-organ failure. Fulminant haemophilus syndrome (HPS) is almost always associated with these patients. Histologically, systemic EBV^+^ T cell lymphoma of childhood is composed of small-sized lymphoid cells that are mild atypia, accompanied by histiocytes phagocytizing red blood cells. Immunohistochemically, the tumor cells of systemic EBV^+^ T cell lymphoma of childhood are mainly positive with CD8 and TIA-1, but different from ENKTL is the negative expression of CD56 (Table 1).^[19,20]^ Primary EBV infections are most common in children and adolescents. If the patient has been infected with EBV for more than 3 months, he is probably considered as chronic active EBV disease (CAEBV).^[20]^ There are more stringent diagnostic criteria:

1. infectious mononucleosis-like symptoms have persisted for more than 3 months;
2. plasma EBV DNA level has been confirmed to be elevated;
3. the involved tissues have characteristic histological manifestations; and
4. EBVR was detected in the involved tissues.^[19,21]^

In addition, CAEBV should be diagnosed in patients without known immunodeficiency, malignancy or autoimmune disorders. The morphologic features of CAEBV are similar to nonspecific inflammation, in which the malignant lymphoproliferation is absent. Immunohistochemically, T-cells are mostly positive with CD4 and CD8, but negative with CD56 (Table 1).

ENKTL is known to be an aggressive malignancy. Multiple studies demonstrated poor prognosis of GI NK/T-cell lymphoma, with the median overall survival of 7 to 9 months.^[4,5]^ At present, surgery and chemotherapy are the common treatment strategies. Kim et al^[5]^ found that patients who underwent postoperative chemotherapy showed a trend of better survival than those treated with chemotherapy alone. In the other hand, the median overall survival of ENKTL in children and adolescents was 12.5 months.^[6]^ Interestingly, Wang et al^[6]^ reported that children and adolescents with early-stage ENKTL treated with primary radiotherapy had a favorable prognosis. Therefore, it is important to accurately and timely diagnose this lymphoma in children and adolescents, which will be beneficial for patients. However, the diagnosis of ENKTL in children and adolescents is challenging.

In summary, ENKTLs in the colon of childhood are very rare and the prognosis of them is poor. Symptoms of this lymphoma are unspecific. The diagnosis based on the combined of morphology, molecular examinations, genetics and clinical manifestation.

## Author contributions

**Conceptualization:** Yi Duan, Juan Huang, ZhiHui Yang.

**Writing – original draft:** Yi Duan.

**Writing – review & editing:** Johannes Haybaeck, ZhiHui Yang.
